# Coupling electro-dewatering and low-temperature air-drying for efficient dewatering of sludge

**DOI:** 10.1038/s41598-021-98477-9

**Published:** 2021-09-27

**Authors:** Hang Lv, Qiao Xiong, Daoguang Liu, Xu Wu

**Affiliations:** 1 School of Environmental Science and Engineering, Huazhong University of Science and Technology, 1037 Luoyu Road, Wuhan, 430074 China; 2 Shanghai Techase Environment Protection Co., Ltd, 1121 North Zhongshan No. 2 Road, Shanghai, 200092 China; 3 College of Environmental Science and Engineering, Tongji University, 1239 Siping Road, Shanghai, 200092 China

**Keywords:** Chemical engineering, Electrochemistry, Environmental chemistry, Green chemistry

## Abstract

This study investigated the effects of electro-dewatering on subsequent low-temperature drying at various potentials and the characteristics of low-temperature air-drying sludge were explored through experiments and multi-physical modeling. Experimental results showed that the extracellular polymeric substance (EPS) content in the sludge was reduced during electro-dewatering process, even the species of organic matter was changed, as well as the dewatered cake tend to form many seepage channels, crack and a certain number of holes. These changes in the properties and structure were conducive to the subsequent low-temperature drying process. For air-drying process, the mass of the sludge cake variation was simulated and results were consistent with the experimental phenomenon. Firstly, the weight of the sludge cake was decreased approximately linearly with time, then tended to stable and reached the dewatering limitation finally. The applied higher electric field intensity (25 V cm^−1^) in the front-end electro-dewatering were conducive to promote water vapor diffusion activity in air-drying stage. Energy consumption and yield analysis results indicated that the combined technology has lower energy consumption and higher yield than that of directly low-temperature drying.

## Introduction

As one of the methods of wastewater treatment, activate sludge process produces large quantities of sludge, which commonly contains over 90 wt% water content and is difficult to be deep dewatered^1–3^. Especially for China, more than 50 million tonne of municipal sludge and over 40 million tonne of organic industrial sludge with 80 wt% water content were produced in 2017^4^. Sludge dewatering is a key link in the existing sludge treatment and disposal process^5–7^. Electro-dewatering and air-drying technology can achieve deep sludge-dewatering (water content less than 60 wt%) without extra agent addition^8,9^, and have already occupied a certain market share in the existing sludge drying market. However, there are few reports on optimum combination of two technologies, especially the effect of electro-dewatering on subsequent low-temperature drying.

Sludge electro-dewatering technology utilizes electric energy to drive electro-migration of water molecules^10,11^. Recently, it was extensively studied^7,12,13^ and also occupied a certain market share. This technology has some excellent benefits, rapid and efficient dewatering, no agent addition, and relatively low operating costs. However, some problems do exist with this approach, mainly including restricting the development or large-scale application, such as electrode corrosion, ohmic heating (Energy dissipation) and high electric resistance at terminal stage of dewatering (Dewatering limits)^14,15^. It was pointed out that the ohmic heat in sludge electro-dewatering process was the main energy loss^16^, so a large number of literatures reported how to reduce the ohmic heat to avoid sludge temperature increment in electro-dewatering process^17,18^. Optimizing parameters with bench-scale, pilot-scale and industrial-scale experiments, Zhang et al. pointed out that when the anode processing capacity was 41 kg m^−2^ h^−1^, energy consumption was controlled at about 70 kWh t^−1^ sludge, and the sludge water content can be reduced to below 60 wt%^19^.

Although sludge water content can be reduced to below 60 wt% by sludge electro-dewatering within 5–20 min, its dewatering limit still restricts further deep dewatering of sludge^9,18^. If the sludge water content was continue to be reduce, the required power consumption would increase dramatically. Low-temperature drying can break through this dewatering limitation and achieve deep dehydration with water content of less than 20 wt%^20^. However, direct thermal dried from 80 to 20 wt% of water content will require much more energy consumption, and relatively low space–time yield^21,22^. There were few reports on the effect of sludge low-temperature air-drying coupled with electro-dewatering and the impact of the front-end electro-dewatering on the back-end further deep air drying.

In China, the existing sludge dewatering field pays too much attention to the indicators of a single technology, and often overlooks the combined and coordinated operation of the entire treatment route^23^. Previous research work found that biochemical action in the aging process changed the EPS composition properties of the sludge and increased the space–time yield of sludge electro-dewatering to 223.46 kg m^−2^ h^−1^^24^. This illustrated that the dehydration characteristics of sludge are closely related to its composition properties^25–27^. The front-end electro-dewatering process would also change the composition of the sludge to a certain extent^13^. The sludge was prone to generate ohmic heat and seepage channels (pores) in the electro-dewatering process^28^. If the two approaches were combined for sludge dehydration, it is worth studying whether the front-end electro-dewatering is beneficial to the back-end low-temperature thermal drying.

The model of sludge drying characteristics can provide guidance for the design of sludge drying equipment and the selection of process parameters. Several studies about drying model can be found. Léonard^26^ established models and investigated the influence of drying air velocity, temperature and environmental humidity on sludge drying performance. It was pointed out that temperature of drying air was the main factor affecting the convective drying. Zhang et al.^29^ established sludge drying system theoretical models for heat pump circulation and moist air circulation, and modeling results were validated with the experimental data. Some researchers also introduced thin-layer drying curve models to simulate the electro-dewatered sludge drying curve^30,31^. However, these models lack the understanding of the time-dependent of sludge air-drying process from perspective of multiphysics and physical geometry. In this paper, the effect of electro-dewatering on subsequent low-temperature drying at various potentials was studied. And this study simulated the time-dependent sludge drying process by introducing porous media model, which considered the air flow field, temperature field, and humidity field comprehensively, and simulated results was validated with low-temperature air-drying experimental data.

## Material and methods

### Sludge sample

Sewage sludge sample was acquired from Shayang wastewater treatment plant (Hubei, China). This wastewater treatment plant adopted oxidation ditch technology to treat wastewater, and the sludge sample was mechanically dewatered by belt press filter. All sample was stored at 4 °C before tests to avoid microbial fermentation after extracting from wastewater treatment plant, and all same group tests were completed within 1 days. Initially the average water content of sludge sample was 81.22 wt% and pH was 7.53.

### Experimental set-up

**Figure 1:**
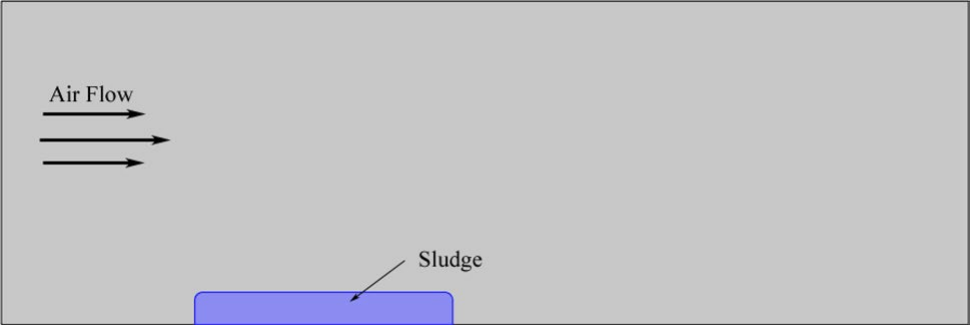
2D Geometric model.

Sludge electro-dewatering experimental test facilities were the same as the previous researches^28^, and the test method for electro-dewatering was also referred to the above approach. The thickness of initial sludge cake was 10.0 mm (about 50.00 g) and electro-dewatered at three different voltage (15, 25, 35 V). All testing for electro-dewatering were conducted on high-power electrochemical workstation (CS150, Wuhan Corrtest Instrument Co. Ltd, China; 0–10 A; 0–50 V). For sludge air-drying tests, they were conducted in a constant temperature control box, and the schematic diagram was similar to Fig. 1. The wind power was provided by a parallel wind electric fan with adjustable speed, and the wind speed can be measured and calibrated with an anemometer. The mass of sludge cake was recorded in real-time through an electronic Bluetooth scale. S-EPS (soluble extracellular polymeric substances) was extracted by centrifugation, the LB-EPS (loosely bound extracellular polymeric substances) and TB-EPS (tightly bound extracellular polymeric substances) contents in sludge were determined by a heat extraction method^32^. The real time water content (*w*) in sludge was calculated as in the following equation:

1 $$w=\frac{{w}_{0}\cdot {m}_{s}-{m}_{t}}{{m}_{s}-{m}_{t}}\cdot 100\mathrm{\%}$$

where *m*_*s*_ represents initial mass of sludge, *w*_*0*_ is initial water content of sludge, and *m*_*t*_ means the real-time mass of water collected or removed water at time *t*.

### Analysis methods

Sludge particle size distribution (from 0.1 to 1000 μm) was tested by a laser particle size analyzer (LPSA), BT-9300ST (Bettersize Instruments Ltd., China). Apparent morphological characteristics of the dewatered sludge were observed by a low-vacuum scanning electron microscopy (SEM, TM3030, Japan). All samples were dried by air at room temperature and sputter coated with gold prior to SEM analysis. And parts of the organic species in EPS was examined by three-dimension exaction and emission matrix (3D-EEM) fluorescence spectrometer (F-4600 FL Spectrometer, Hitachi, Japan). The above analysis and characterization methods refer to the previous research and literatures^33^.

### Numerical modelling

After electro-dewatered, transient change process model of sludge air-drying weight was set up by using COMSOL Multiphysics simulation software (No. 9401017). As shown in Fig. 1, the 2D geometry was built to represent the air-drying scene in the model, which included both sludge and wind field. The initial input parameters of the model were shown in Table 1.

**Table 1:** Initial values and some parameters setting for simulation.

Parameter	Value	Meaning
*P*_*0*_	1 atm	Ambient pressure
*T*_*0*_	25 °C	Ambient temperature
*u*_*0*_	1.5 m s^−1^	Free stream velocity
*D*_*wa*_	2.6 * 10^–5^ m^2^ s^−1^	Water–air diffusivity
*H*_*vap*_	44,172 J mol^−1^	Heat of vaporization
*K*	1000 s^−1^	Evaporation rate^35^
*c*_*0*_	0.51 mol m^−3^	Initial concentration

Evaporation in porous media was an important process in sludge air-drying. Many physical effects must be considered: fluid flow, heat transfer and transport of participating fluids and gases. All of these effects were strongly coupled and can be implemented by using the predefined interfaces of COMSOL Multiphysics. The thermodynamic properties of air with water vapor can be described by mixture laws based on the amount of water vapor and dry air. This process was accomplished automatically when moist air was selected as fluid type, and the governing equations was built in the *Heat Transfer Module*. The concentration *c* (mol m^−3^) from the transport equation was used as water vapor input. Heat transfer in fluids and sludge cake (porous media) were described as following equations:

2 $$\uprho {C}_{p}\frac{\partial T}{\partial t}+\rho {C}_{p}u\cdot \nabla T+\nabla q=Q$$

3 $$\mathrm{q}=-\mathrm{k}\nabla \mathrm{T}$$

ρ (SI unit: kg m^−3^) was the density.

*C*_*p*_ (SI unit: J kg^−1^ K^−1^) was the heat capacity at constant pressure.

*k* (SI unit: W m^−1^ K^−1^) was the fluid thermal conductivity.

u (SI unit: m s^−3^) was the fluid velocity field.

*Q* (SI unit: W m^−3^) was the heat source. In this model, the evaporation of water in sludge cake would absorb heat, and Q was calculated in Eqs. (2–4).

4 $$\mathrm{Q}={H}_{vap}\cdot {m}_{vap}$$

where *H*_*vap*_ (J mol^−1^) was the latent heat of evaporation, and *m*_*vap*_ (mol m^−3^ s^−1^)) was the mass of water evaporated, which was calculated with Eqs. (2–6). For the evaporation process, the evaporated mass of water was added as source term in the transport equation. Evaporation occurred if the concentration of water vapor was below the equilibrium concentration, which was determined by the saturation concentration *c*_sat_.

5 $${c}_{sat}=\frac{{p}_{sat}\left(T\right)}{RT}$$

With the saturation pressure *p*_sat_ and the ideal gas constant R = 8.314 J mol^−1^ K^−1^).

6 $${m}_{vap}=K\cdot \left({c}_{sat}-c\right)$$

where *K* (s^−1^) was the evaporation rate, and *c* was the current concentration. The evaporation rate depended on the material properties and the process that caused it. This corresponded to the assumption that vapor and liquid was in equilibrium, in other words, the time scale for evaporation was much smaller than the smallest time scale of the transport equations^34^.

The diffusion and migration process of water vapor was described by the following formula.

7 $$\nabla \cdot \left(-D\nabla c\right)+{\varvec{u}}\cdot \nabla c={R}_{w}$$

8 $${R}_{w}={m}_{vap}$$

where *D* represented the water vapor diffusion coefficient, ***u*** was velocity of the wet air, and *R* was the reaction rate, which was actually the water vapor evaporation rate. In sludge porous media, the diffusion rate of water vapor in the moist air gradually slowed down as the water content decreased. In the porous domain, the diffusivity of water vapor in sludge (*D*_*e*_ (m^2^ s^−1^)) can be adjusted by the diffusivity of water vapor in air (*D*_*wa*_ (m^2^ s^−1^)) according to the following formula.

9 $${D}_{e}={D}_{wa}.\alpha \left(w\right)$$

where α(*w*) was the water vapor diffusion activity in the sludge cake, which was the interpolation function obtained in the experiment.

The laminar Navier–Stokes equation was used to model the fluid flow from the air domain. Brinkman equation was used for porous domain, and the Laminar Flow Interface was expanded by enabling porous media domains. The coupling of both flow regimes was done automatically in this way. The resulting velocity field then can be used to model convective heat and species transport.

## Results and discussion

### Effect of coupling sludge electro-dewatering and low-temperature air-drying

**Figure 2:**
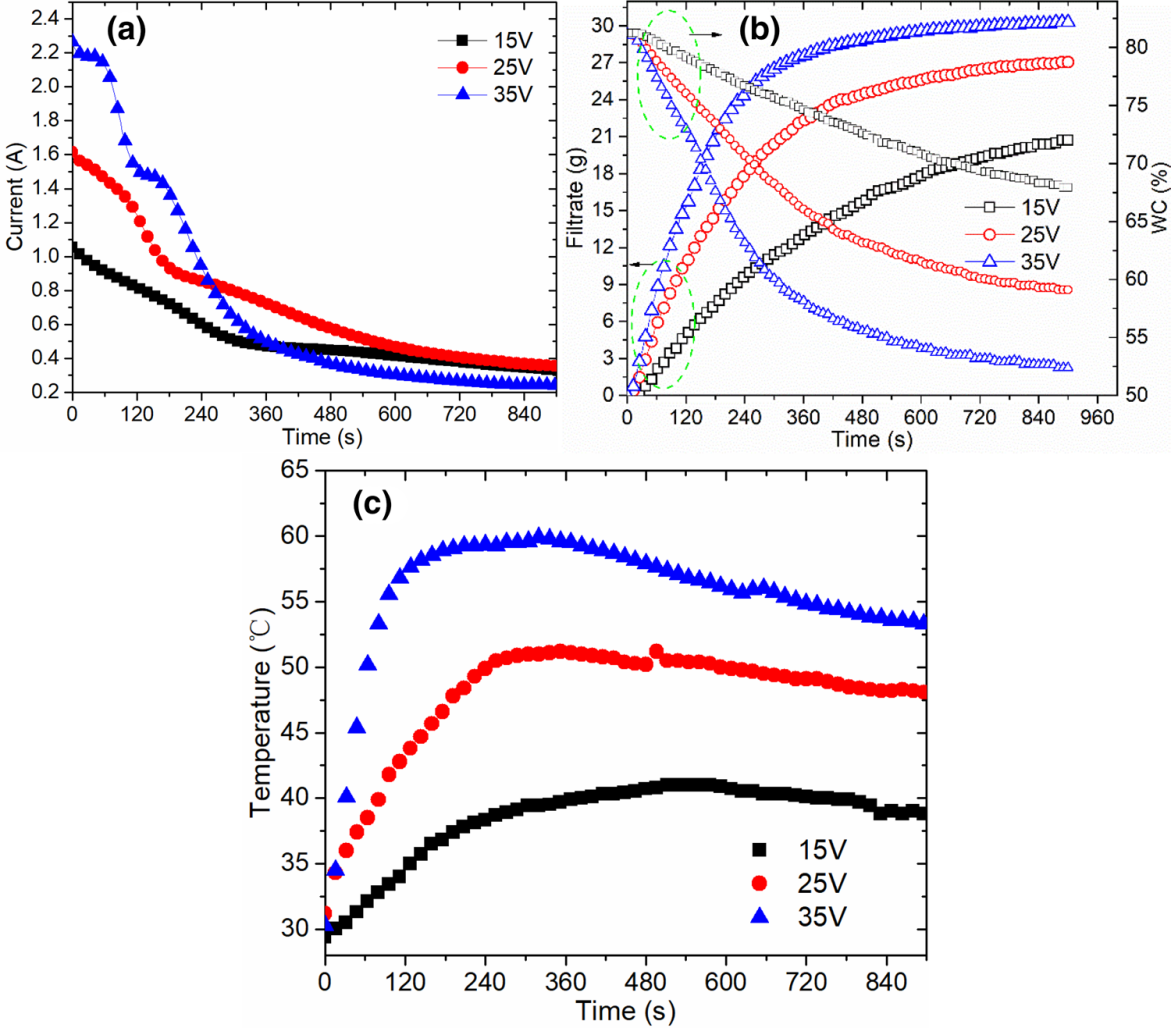
(**a**) Current, (**b**) filtrate, and (**c**) temperature variation in different voltage electro-dewatering process.

**Figure 3:**
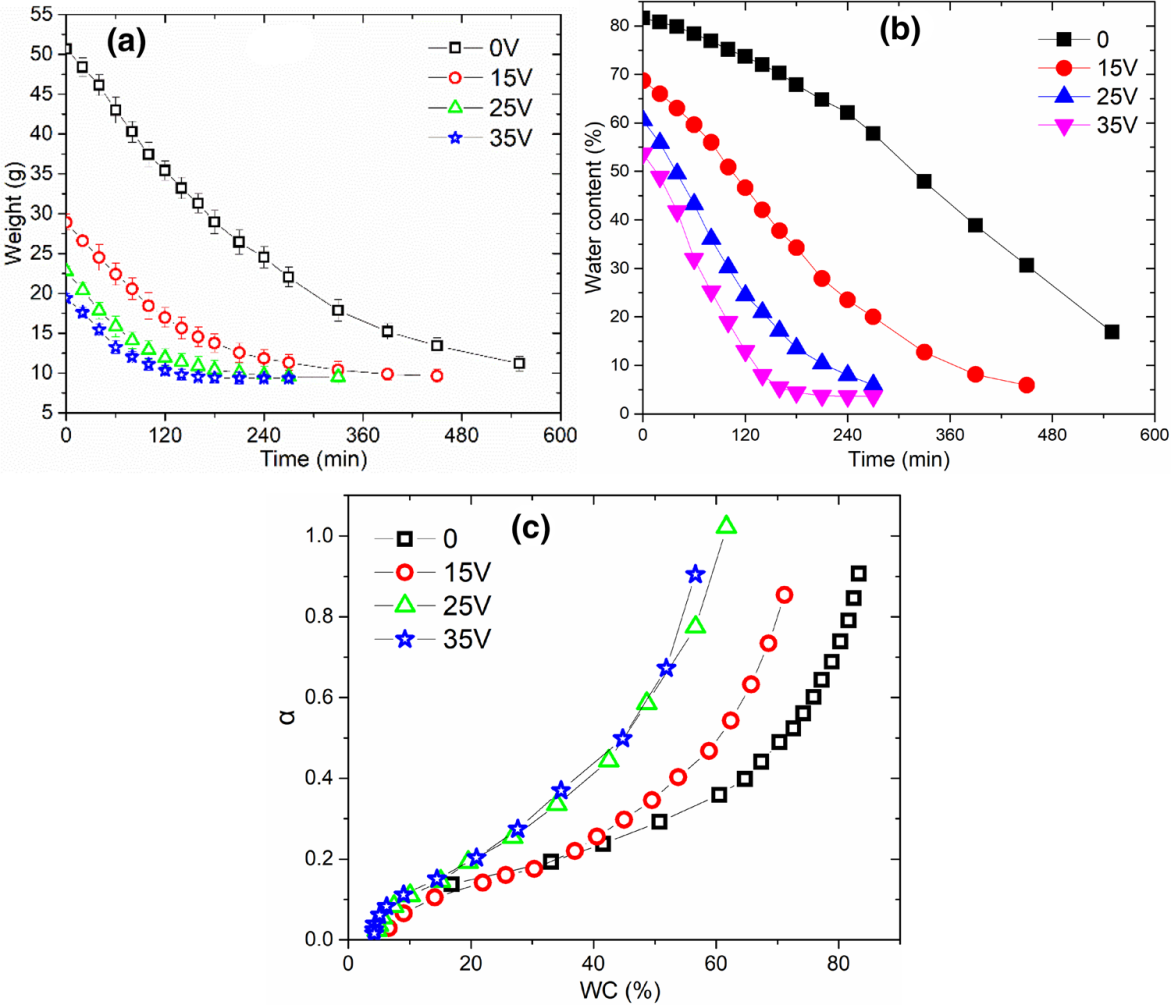
(**a**) The weight of sludge cake, (**b**) water content variation during air-dry process and (**c**) the water vapor diffusion activity in sludge cake (the ratio of the slope of the sludge air-drying curve to the slope of the water–air drying curve under the same conditions).

It can be seen from Fig. 2 that the variation trends of current, filtrate and temperature in the process of sludge electro-dewatering were consistent with the phenomenon observed in the existing literatures. And the dewatering process could be divided into three stages (rapid, slow and limit dewatering stage). In the rapid dewatering stage, the mass of filtrate increased almost linearly with time, then entered the slow dewatering stage, and finally gradually transited to the limit dewatering stage with almost no filtrate. The greater the electric field strength, the lower the final water content in sludge, and more energy consumption was needed (Table 2). For example, under the conditions of 15 V, 25 V, and 35 V, the limit of sludge electro-dewatering was reached at about 900 s, 700 s and 500 s respectively, and the final water content of dewatered sludge cake was 67.92 wt%, 59.10 wt%, and 52.36 wt%, respectively. Even if the dewatering time was extended, it was difficult to continue to deepen the degree of dehydration. However, low temperature air drying technology can further dry the sludge. After electro-dewatering, the air-drying mass variation curve of sludge cake was showed at Fig. 3 (Temperature: about 45.0 °C, Relative humidity: about 40.0%). It can be seen that the mass of sludge cake decreased rapidly in the early stage, and then as sludge water content reduced, this trend (the air-drying mass of the sludge cake decreased) became more slowly. For example, during the first 120 min for original sludge (0 V), air-drying process reduced the mass of the sludge cake by about 15.0 g, and the mass of the sludge cake was only reduced by about 11.0 g from 120 to 240 min. This phenomenon also existed in electro-dewatered sludge air-drying process. The lower the water content of the sludge, the slower the air-drying mass decline rate. At the same time, air-drying also existed certain limitations. Under current experimental conditions (Temperature: about 45.0 °C, Relative humidity: about 40.0%), the limit water content of sludge after air-drying was about 5.0 wt%.

**Table 2:** Energy consumption and time–space yield by electro-dewatering.

	Y_70_	Y_65_	Y_60_	E_70_	E_65_	E_60_
15 V	59.70	–	–	36.47	–	–
25 V	155.64	100.75	54.79	47.14	63.81	89.99
35 V	279.72	188.67	133.33	58.74	79.34	95.61

Y_70_, Y_65_,Y_60_ were represent time–space yield when water content reach 70, 65 and 60 wt% respectively, unit: kg m^−2^ h^−1^. E_70_, E_65_, E_60_ were represent electric energy consumption per tonne sludge when water content reach 70, 65 and 60 wt% respectively, unit: kWh t^−1^.

After electro-dewatering under different electric field strength conditions, the air-drying water content variation curve of sludge cake was showed as Fig. 3b (Temperature: about 45.0 °C, Relative humidity: about 40.0%). After a certain intensity of electro-dewatering, the sludge cake was easier to air dry. For example, it took about 300 min to air-dry the original sludge directly to a water content of 60 wt%. And it also took about 220 min to realize deep dewatering, which reduced water content of 60 wt% to 20 wt% by air drying. However, electro-dewatered sludge can be directly air-dried under 25 V, and water content dropped from 60 to 20 wt%. This process took only about 140 min, and 80 min less than the previous 220 min of sludge air-drying. When 35 V voltage was applied for sludge electro-dewatering, it can directly reduce the water content to about 55 wt% within 15 min, and then air-drying to 20 wt% water content took about 100 min, while this process (air dried sludge water content from 55 to 20 wt%) for original sludge (0 V) need 200 min. Under the same air drying process, it took about 170 min to dewater sludge with15 V. It can be seen from Fig. 3c that the water vapor diffusion activity in sludge cake gradually decreased as sludge water content reduced. Under the same water content, the diffusion activity of electro-dewatered sludge at 25 V and 35 V was significantly higher than that of the original sludge. The above phenomenon indicated that higher electric field strength applied for sludge electro-dewatering would be more conducive to improve yield efficiency of subsequent sludge low-temperature air drying.

### Changes of sludge EPS after electro-dewatering

**Figure 4:**
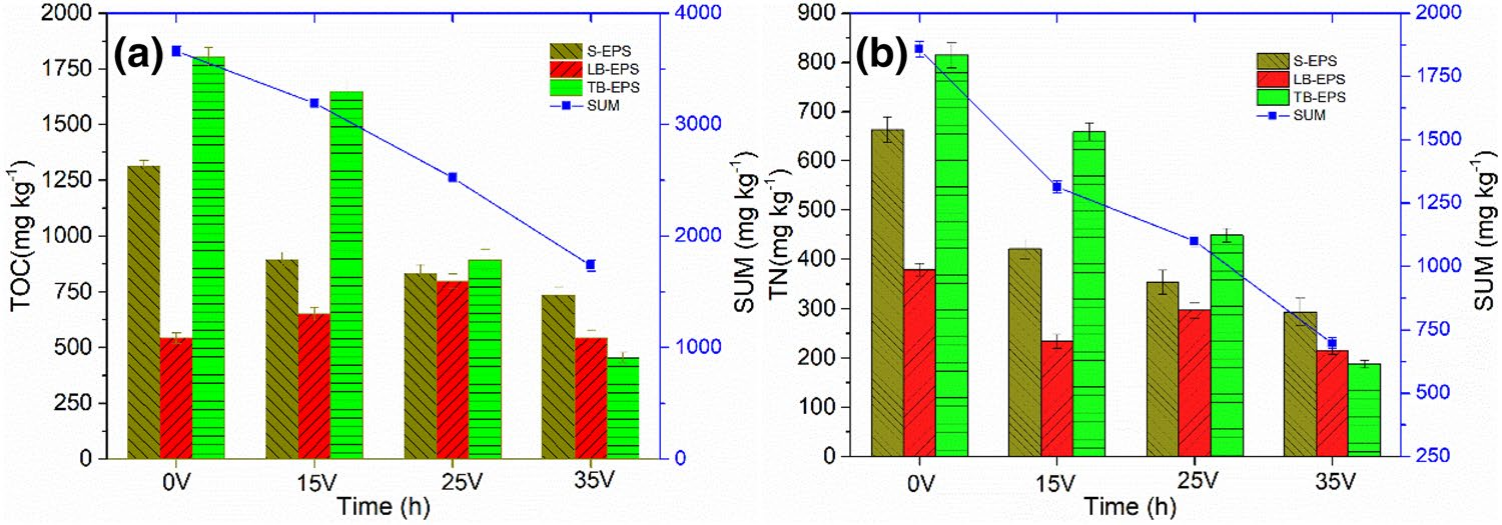
(**a**) TOC and (**b**) TN content in sludge after electro-dewatering.

Sludge properties may undergo certain changes during electro-dewatering process. Figure 4 showed the difference in sludge extracellular polymer (EPS) content for electro-dewatering at different voltages. It can be seen from the Fig. 4 that the total amount of EPS in electro-dewatered sludge decreased continuously with the increasing of the applied electric field intensity. Actually, in the electro-dewatering process, part of the dissolved organic matter in sludge would be transferred to the filtrate along with the water molecules electro-osmotic flow. On the one hand, the increasing of the electric field intensity contributed to more electro-osmotic flow, resulting in an increasing in the amount of water and organic matter removed from sludge; On the other hand, the 35 V voltage condition would cause some EPS rupturing, leading to more organic matter release into the liquid phase during the electro-dewatering process^28^. The total organic carbon (TOC) content was reduced from about 3661 mg kg^−1^ of the original sludge to about 1733 mg kg^−1^ of 35 V electro-dewatered sludge, and the total nitrogen (TN) content was reduced from about 1857 mg kg^−1^ to about 697 mg kg^−1^ of the 35 V dewatered sludge. Among them, the content of TB-EPS decreased most obviously. As the applied voltage increased, its content gradually decreased from about 1805 mg kg^−1^ in original sludge to 456 mg kg^−1^ in the 35 V electro-dewatered sludge cake, with a decrease of more than 74.00%. As reported in literatures, the bound water content has a strong relationship with TB-EPS^36^. The decrease of TB-EPS content may contribute to reducing the binding energy between bound water and sludge flocs, which may be beneficial to subsequent air drying effect.

**Figure 5:**
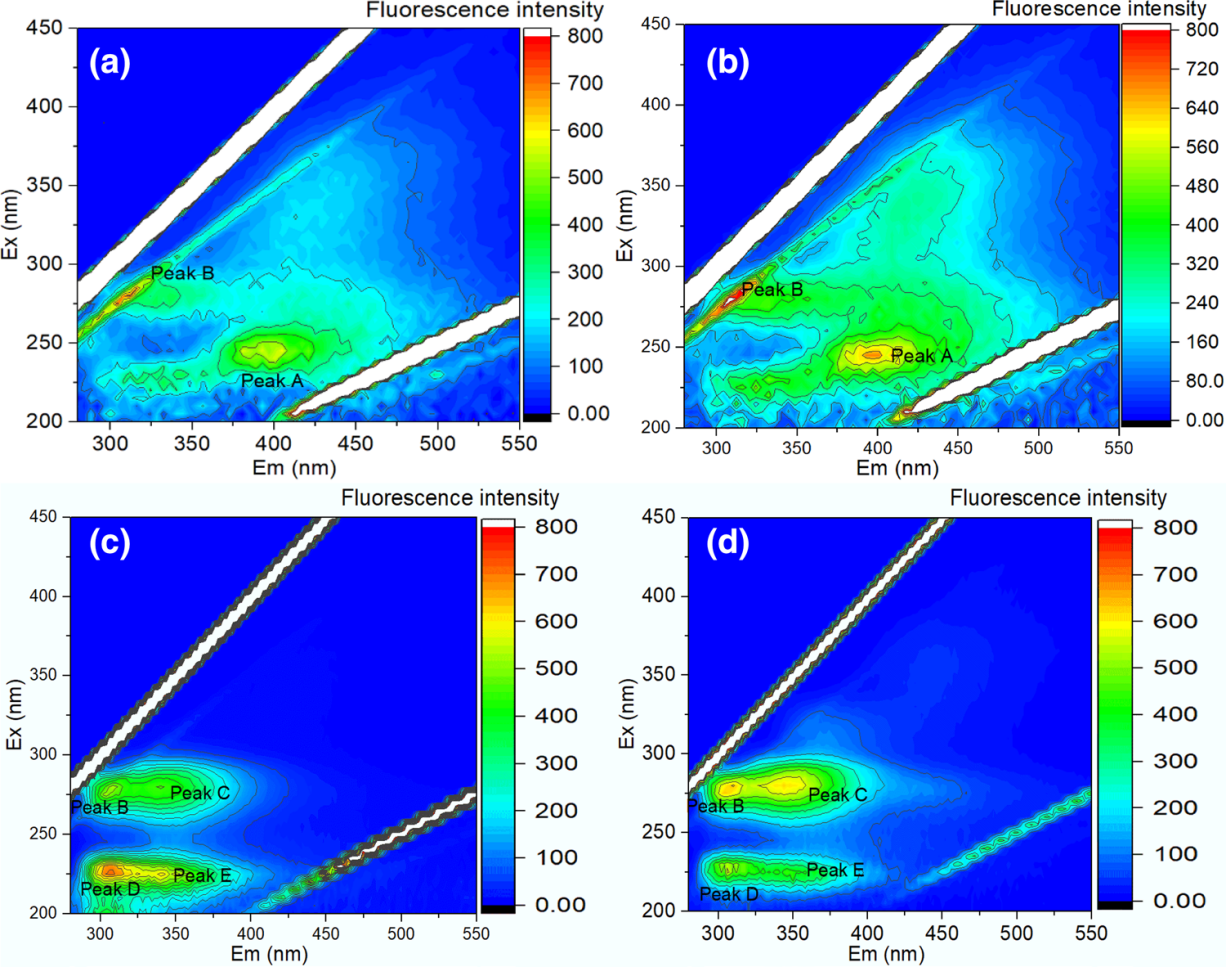
3D-EEM spectra of EPS extracted from (**a**) initial sludge and (**b**) 15 V, (**c**) 25 V, (**d**) 35 V dewatered sludge.

After a certain voltage of electro-dewatering, not only the EPS content in sludge changed, but also some organic components in the EPS varied. The three-dimensional fluorescence spectrum was performed to analyze organic component in EPS, and results were displayed in Fig. 5. It was obvious that two separate peaks A and B appeared in the EPS extracted from original sludge, which were located in the ranges of Ex/Em 240–245/385–390 nm and Ex/Em 280–285/310–315 nm. In the light of literature recorded^37^, these two peaks of organic substance represent Fulvic acid-like and tyrosine and protein-like substance respectively. Electro-dewatering at 15 V, there were still only two peaks A and B appeared in sludge EPS 3D-EEM fluorescence spectrum. Notably, there were 4 peaks appearing at Fig. 5c,d for 25 V and 35 V electro-dewatered sludge. Compared with another two Figures (Fig. 5a,b), three new peaks of C, D, and E appeared, and the previous peak A disappeared. This newly appeared peaks of C, D and E were located at Ex/Em 275–280/340–345 nm (Tryptophan), Ex/Em 220–225/310–315 nm (Aromatic Protein I) and Ex/Em 220–225/340–345 nm (Aromatic Protein II) respectively. When applied voltage reached a certain level, the maximum temperature of sludge cake in the process of electro-dewatering could exceed 50 °C, which may cause some of the protein or polysaccharides in the EPS to be denatured or decomposed, and even cause some cells to rupture^28,38^. This phenomenon may cause sludge particles to release part of the bound water or reduce the bound energy between water and sludge flocculation particles^24,27^.

### Changes of sludge apparent properties after electro-dewatering

**Figure 6:**
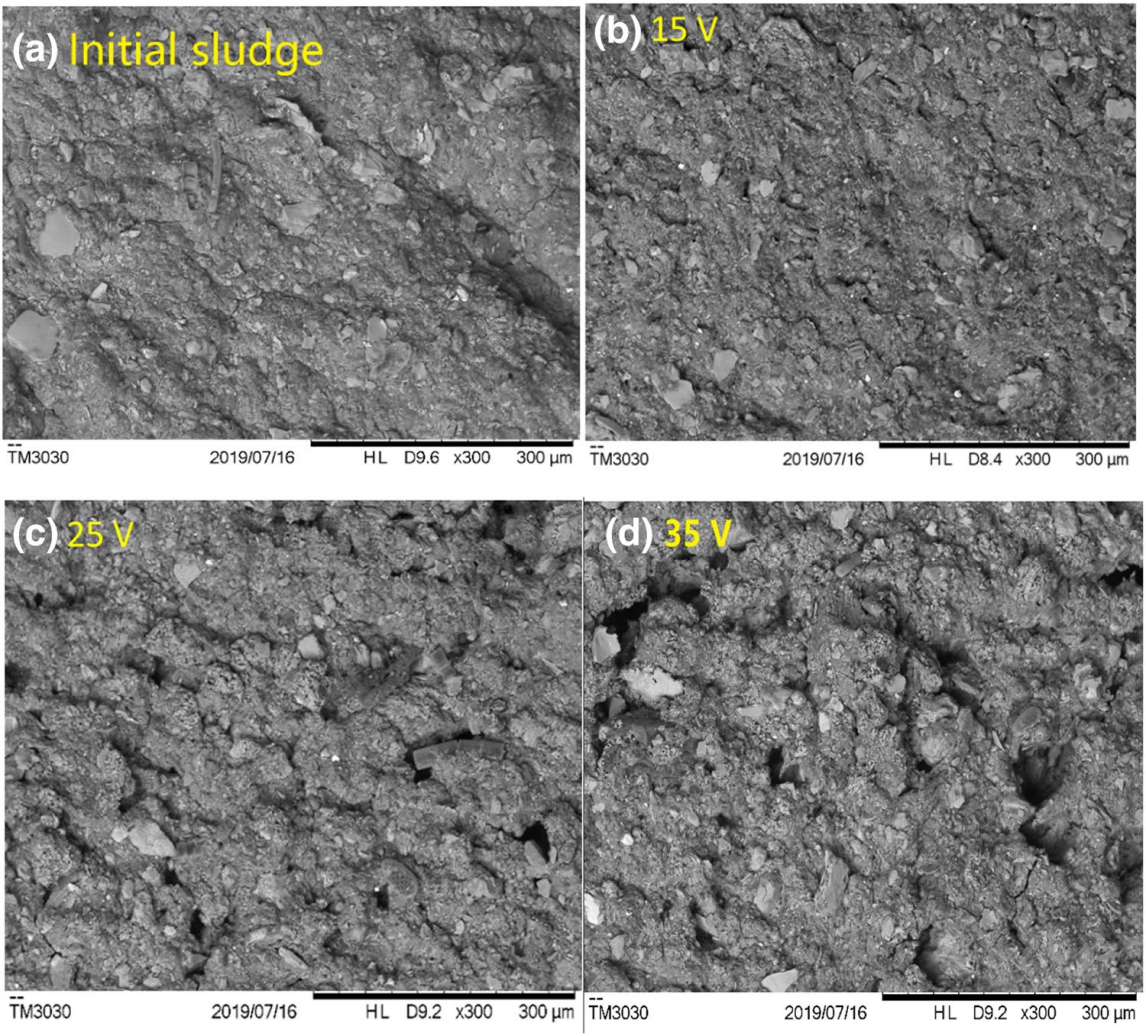
SEM micrographs of electro-dewatering sludge under different voltages.

**Figure 7:**
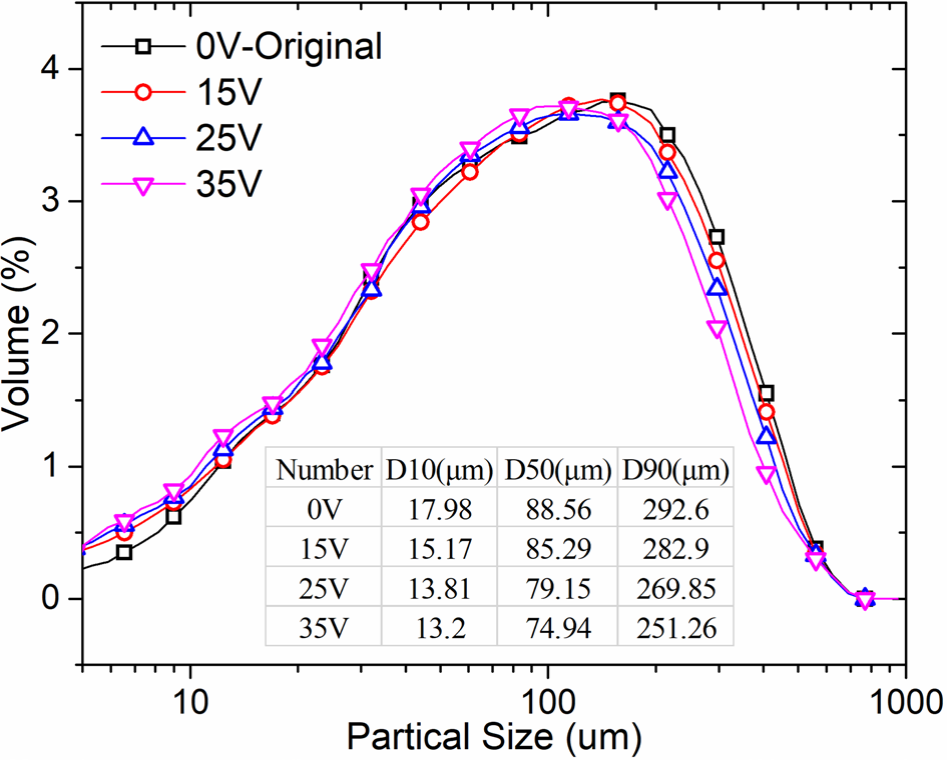
Sludge particle size distribution.

It not only caused variation in the composition and content of EPS, but may also lead to changes in internal structure and surface morphology. The surface morphology of the sludge cake after electro-dewatering at different voltages was shown in Fig. 6. It can be seen that the surface morphology of the original sludge was compact, and almost without cracks and voids, which was the same as that of 15 V electro-dewatered sludge. However, cracks and small holes appeared on the surface of the sludge after 25 V and 35 V electro-dewatering. This phenomenon was consistent with the EPS change results in Fig. 5 and the particle size distribution results in Fig. 7. Although the particle size distribution of the original sludge in Fig. 7 was similar to that of the sludge cake after electro-dewatering, there were still some differences between each other. For example, the median particle size of the original sludge and the sludge cake after 15 V electro-dewatering were 88.56 μm and 85.29 μm, while the median particle size of the sludge cake electro-dewatered by 25 V and 35 V was 79.15 μm and 74.94 μm respectively, a slight decrease compared to the previous two. This phenomenon indicated that when the applied voltage reached a certain degree, some organic matter may be changed under the electric field and the temperature field generated by Joule heat, and some of organic matter flowed out during the electro-dewatering process, which reduced the viscosity of the sludge and make the sludge cake easier to crack. At the same time, a seepage channel was formed. These cracks and small holes structures increased the surface area in contact with air, which was more conducive to subsequent air drying than the compact structure of the original sludge.

### Simulation results and key performance indicators analysis

**Figure 8:**
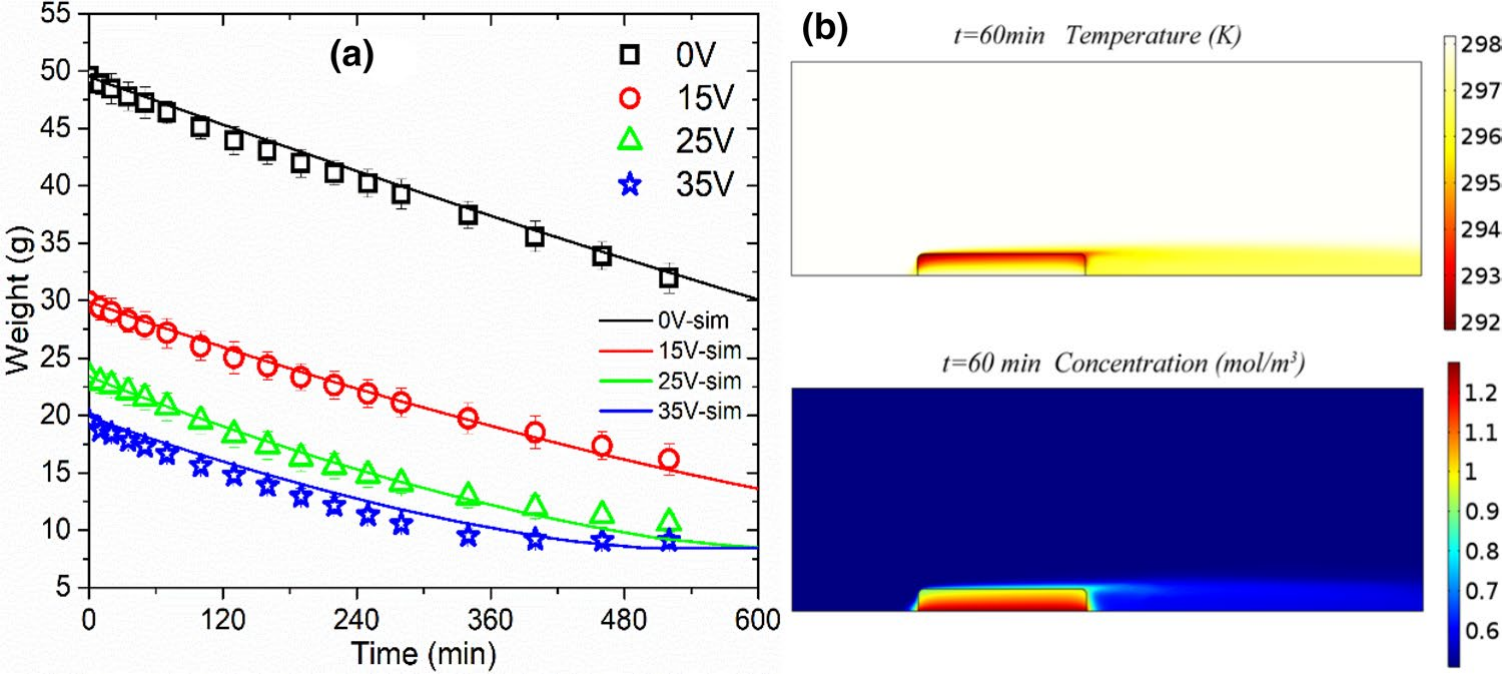
After electro-dewatering, (**a**) sludge air-drying mass curve, simulated and verified by experiment. (**b**) Simulated water vapor concentration and temperature distribution at 60 min (35 V).

In order to verify the validity of the model, sludge air-drying experiment was carried out at room temperature, and the simulation results for the mass of sludge were compared with the experimental data. It can be seen from Fig. 8a that the simulation results were consistent with the experimental phenomenon. The evaporation of water in the sludge caused the distribution of the temperature field, and the diffusion led to the distribution of the water vapor concentration field (Fig. 8b). The weight of sludge cakes decreased linearly in the early stage, and gradually slowed down in the later stage until it reached the dewatering limit. The linear declined in the early stage, which may be because the water transport rate in the sludge was greater than the evaporation rate. The later nonlinearity stage may be due to the fact that the transport or diffusion rate of water vapor in porous sludge was less than the rate of diffusion into the air. At the same time, it can be observed that the sludge after electro-dewatering was more conducive to the subsequent low-temperature air drying process (sludge air-drying weight and water content decreased faster). In addition, sludge could shrinkage during air-drying process, which would inevitably have a certain impact on the drying efficiency. With the shrinkage of the sludge cake, the diffusion rate of water vapor in the sludge medium would decrease. This factor is described in the model by the molecule diffusion activity α(*w*).

It can be seen from Table 3 that if one tonne of sludge with water content of 81.22 wt% was directly air-dried to 10.0 wt%, the required energy consumption was about 292.53 kWh. And if the electro-dewatering technology was applied to firstly dewatering to about 60.0 wt%, then the air-drying method was used to dry to 10.0 wt% water content, the energy consumption was only about 199.94 kWh (Reference energy consumption for thermal drying: 0.40 kWh kg^−1^, and electro-dewatering E60 in Table 2: 95.61 kWh t^−1^ sludge). For the analysis of the yield, it took over 550 min if the sludge directly air-dried from water content of about 81.22 wt% to 10.0 wt%. And sludge electro-dewatered first to water content of about 60.0 wt%, then air-dried to 10.0 wt%, the total time required was less than 140 min. The less time it took, the more sludge can be processed per unit time. Therefore, it can be found that the combination of electro-dewatering coupled with low-temperature drying technology has advantages in terms of energy consumption and productivity.

**Table 3:** Energy consumption by thermal drying in literature.

Author	Material	Energy consumption/kWh kg^−1^ (based on removed water)	Refs.
Zhou et al.	Waste activated sludge	1.20	^39^
Hong et al.	Waste activated sludge	0.40	^40^
Eom et al.	Sludge	Over 0.50	^41^

## Conclusions

Under a certain electric field strength, the EPS content in sludge cake decreased, the species of organic matter also changed during the electro-dewatering process and the dewatered cake tended to form crack and a certain number of holes. Changes in the content and composition of EPS in sludge cake may reduce the binding energy of the bound water, and the cracks and holes formed in sludge cake were beneficial to increase the contact area with the air during the subsequent air-drying process. The simulation results of the model were consistent with the experimental phenomena, indicating that the multi-physical model was suitable for the drying characteristics of electro-dewatering sludge. Taking advantage of the changes in the properties and structure of the sludge cake after the front-end electro-dewatering, followed by low-temperature drying, was more conducive to increasing the space–time yield of the overall sludge treatment and reducing the energy consumption of the overall treatment.
